# Coverage and contextual factors associated with insecticide-treated net use among women of reproductive age in Nigeria: evidence from the 2021 malaria indicator survey data

**DOI:** 10.1371/journal.pgph.0004207

**Published:** 2025-02-18

**Authors:** Amadou Barrow, Oluwakemi Christie Ogidan, Chimezie Igwegbe Nzoputam, Michael Ekholuenetale

**Affiliations:** 1 Department of Public and Environmental Health, School of Medicine and Allied Health Sciences, University of The Gambia, Kanifing, Gambia,; 2 Department of Epidemiology, College of Public Health and Health Professions, University of Florida, Gainesville, Florida, United States of America,; 3 Department of Nursing Science, College of Medicine, Ekiti State University, Ado Ekiti, Ekiti State, Nigeria,; 4 Department of Medical Biochemistry and Molecular Biology, School of Basic Medical Sciences, University of Benin, Benin City, Nigeria,; 5 Department of Public Health, Center of Excellence in Reproductive Health Innovation, College of Medical Sciences, University of Benin, Benin City, Nigeria,; 6 Department of Epidemiology and Medical Statistics, Faculty of Public Health, College of Medicine, University of Ibadan, Ibadan, Nigeria,; 7 Faculty of Science and Health, School of Health and Care Professions, University of Portsmouth, Hampshire, United Kingdom; University of Alabama at Birmingham, UNITED STATES OF AMERICA

## Abstract

Nigeria continues to face a serious public health problem due to malaria, which contributes around 27% of all cases worldwide. Although insecticide-treated nets (ITNs) are essential interventions for preventing malaria, their use in Nigeria is still sub-optimal. Understanding the factors influencing ITN use is essential to improve malaria control strategies. This study aimed to investigate the prevalence of and factors associated with ITN use among women of reproductive age in Nigeria. We conducted a secondary data analysis of the 2021 Malaria Indicator Survey (NMIS). The sample included 14,476 women of reproductive age (15–49 years) from 567 clusters across Nigeria’s six geopolitical zones. Multilevel logistic regression was used to examine the contextual factors associated with ITN use, accounting for both fixed and random effects. Statistical significance was set at p<0.05, with 95% confidence intervals reported for all estimates. The weighted prevalence of ITN use was 41.5% (95% CI: 39.7, 43.3). The results showed Muslim women had 21% (aOR= 0.79; 95% CI: 0.67–0.94) lower odds of ITN use, when compared with the Christian women. Those who had living children were more likely to report ITN use, when compared with women with no child. The non-poor women had higher odds of ITN use, when compared with the poor women (aOR= 1.35; 95% CI: 1.19–1.52). In addition, women from multi-ethnic had 26% lower odds of ITN use, when compared with those from mono-ethnic community (aOR= 0.74; 95% CI: 0.57–0.94). The geographical region was significantly associated with ITN use among Nigerian women. Those from high-level community poverty had 53% reduction in the odds of ITN use, when compared with women from low-level community poverty (aOR= 0.47; 95% CI: 0.32–0.68). ITN use among women of reproductive age in Nigeria remained below the national target. Interventions to increase ITN use should consider the complex interplay of individual and community-level factors. Targeted strategies addressing socioeconomic disparities, leveraging cultural contexts, and implementing community-based approaches are crucial for improving ITN utilization and reducing the malarial burden in Nigeria.

## Introduction

Malaria remains a critical global health challenge, with sub-Saharan Africa bearing a disproportionate burden globally. In 2021, the region accounted for approximately 95% of 247 million global malaria cases and 96% of 619,000 malaria-related deaths [1]. Nigeria, in particular, has the highest malaria burden, representing approximately 27% of global cases and 32% of deaths [2]. This impact extends beyond health, imposing a significant economic burden on Nigeria, estimated at approximately 1.1% of the country’s GDP [3]. Women of reproductive age are especially vulnerable and face increased risks of severe anemia, maternal mortality, and adverse birth outcomes [4,5]. In malaria-endemic areas, this demographic group experiences a higher infection prevalence compared to non-pregnant women and adult men [6,7]. The consequences are severe, with malaria contributing to an estimated 11% of maternal mortality in Nigeria, highlighting its devastating effects on pregnant women [8,9]. Insecticide-treated nets (ITNs) have proven to be one of the most cost-effective interventions for malaria prevention, capable of reducing malaria incidence by up to 50% in endemic areas and all-cause child mortality by approximately 20% [10].

Recognizing the effectiveness of ITNs, global and regional policy frameworks have emphasized increasing ITN coverage and use. The United Nations Sustainable Development Goals (SDGs), particularly SDG 3.3, call for ending the malaria epidemic by 2030 [11]. The World Health Organization’s Global Technical Strategy for Malaria 2016–2030 aims to achieve a 90% reduction in malaria incidence and mortality rates globally compared to 2015 levels [12]. At the regional level, both the African Union’s Agenda 2063 and the Roll Back Malaria Partnership’s Action and Investment to Defeat Malaria 2016–2030 emphasize the critical role of ITNs in malaria control efforts [13,14]. Despite these commitments and substantial investments in ITN distribution, utilization rates remain suboptimal. The World Malaria Report 2022 indicated that while 65% of households in sub-Saharan Africa owned at least one ITN, only 40% of the population had access to an ITN within their household and 33% slept under an ITN the night before the survey [1]. In Nigeria, the situation mirrors this trend, with the 2018 Demographic and Health Survey reporting 61% household ownership, 52% population access, and only 36% use the night before the survey [15]. This discrepancy between ownership and use highlights the complex nature of ITN utilization, which is influenced by various individual, household, and community-level factors [16].

Previous studies have identified several determinants of ITN use, including socioeconomic status, educational level, household size, and perceived malaria risk [17]. A systematic review of studies from sub-Saharan Africa found that wealthier households, those with higher education levels, and those with pregnant women or young children were more likely to use ITNs [18]. However, the relative importance of these factors may vary across different contexts within Nigeria, given the country’s diverse socio-demographic landscape [19–21]. Community-level factors, such as social norms, ethnic diversity, and overall community development, have been increasingly recognized as crucial influences on health behaviors, including ITN use [22]. A multi-country analysis of 29 sub-Saharan African countries found that community-level factors accounted for up to 40% of the variation in ITN use [23]. Women of reproductive age play a pivotal role in household decision-making regarding health practices, including ITN use [24]. In many African settings, women are primarily responsible for hanging and maintaining ITNs, as well as ensuring that family members, particularly children, sleep under them [25]. Understanding the factors associated with ITN use in this demographic is crucial for designing targeted interventions and improving overall malaria prevention strategies.

This study aims to address the knowledge gap in recent nationally representative studies examining the multilevel determinants of ITN use among women of reproductive age in Nigeria [26]. This research is particularly significant given Nigeria’s diverse ethnic, cultural, and socioeconomic landscapes, which may influence ITN use patterns differently across various regions and communities. By addressing these critical issues, our study contributes to the broader goal of malaria elimination and improved maternal and child health outcomes in Nigeria and other similar settings.

## Methods

### Data source

We used individual woman questionnaire data from the 2021 Nigeria Malaria Indicator Survey (NMIS). In total, 14,476 women of reproductive age (15–49 years) made up the study’s sample that was analyzed. The data collection took place from 12 October to 4 December 2021. The majority of survey indicators for the entire country, for urban and rural areas separately, and for each of the six geopolitical zones in the country, which comprise 36 states and the Federal Capital Territory (FCT) were included in the sample for the 2021 NMIS.

### Sample design

The sample frame for the Federal Republic of Nigeria’s projected 2023 Population and Housing Census (PHC) was utilised in the 2021 NMIS. Nigeria is separated into states administratively. Local government areas (LGAs) are the lowest level of governance in each state. Within LGAs are wards, and within wards are localities. Census enumeration areas (EAs), which are handy areas, are further subdivided into localities. Based on the EAs for the projected 2023 PHC, the primary sampling unit (PSU), also known as a cluster unit for the 2021 NMIS, was defined. For the NMIS of 2021, a two-phase sampling approach was chosen. A probability proportional to the EA size was used to choose 568 EAs in the first stage.

The number of households inside an EA determines its size. The sample was chosen in a way that made it representative of every state. As a consequence, there were 568 clusters nationwide - 195 of which were in urban areas and 373 of which were in rural areas. Between August 26, 2021, and September 18, 2021, all of the households in these clusters were listed in full. The lists of homes that were produced were used as the sample frame to choose the households for the second stage. In the 2021 NMIS sample, GPS dongles were utilised to record coordinates during the households listing process [27]. By using equal probability systematic sampling, 25 households from each cluster were chosen for the second step of the selection procedure. The datasets are available in the public domain via https://dhsprogram.com/data/dataset/Nigeria_MIS_2021.cfm?flag=1.

### Selection and measurements of variables

#### Outcome variable.

The outcome variable in the study was ITN use. It was derived from the question variable “*V461 - Respondent slept under mosquito bed net*”. This yielded a binary response, with ‘yes’ coded as “1” indicating that the respondent slept under ITN the night before the survey, and ‘no’ coded as “0” indicating otherwise. This follows the operational definition of ITN use as used by NMIS [27]. The MIS 2021 provides current and comprehensive data on malaria-related indicators, including ITN use.

#### Explanatory variables.

Previous studies provided the basis for the factors this study examined [28–30]. Age (in years): 15–24, 25–34, 35–49; Education: No education/primary, Secondary/higher; religion: Christianity, Islam, Others; exposed to malaria messages: no, yes; number of living children: 0, 1–2, 3–4, 5+; wealth: poor, non-poor; sex of household head: male, female; region: North Central, North East, North West, South East, South South, South West; place of residence: urban, rural; community-level ethnic: mono-ethnic, multi-ethnic; community-level poverty: low, medium, high; community-level education: low, medium, high; community-level exposure to malaria messages: low, medium, high.

#### Analytical approach.

Stata software version 17.0 (Stata Corporation, College Station, Texas, USA) was used for data analysis. Since the study included the multi-stage stratified cluster sample design, we employed the survey module’s (‘svy’) function to account for sampling design (weighting, clustering, and stratification). Percentage was employed in the univariable analysis. The fixed and random effects of ITN use were investigated using the multilevel multivariable binary logistic regression. In order to assess multicollinearity, which is known to raise serious issues with the logit model, the variance inflation factor was employed [31].

We designed a two-level model for binary response reporting ITN use, at level 1 for individual women factors nested within communities and level 2 for community/EA-level factors. We built four models. First, the community-level variance was computed in the empty or unconditional model with no explanatory factors. We utilised the null or empty model as the benchmark to calculate the extent to which community characteristics may account for the observed changes. We used the results to justify the multilevel statistical model. Since the variance was statistically significant, the use of multilevel regression was established. The second model included the individual-level factors, the third model included the community-level factors. Finally, the fourth model (full model) adjusted for the individual and community-level factors. The level of significance was determined at p < 0.05. To choose the best model from the four models, the Bayesian and Akaike Information Criteria were used. A lower Akaike or Bayesian Information Criterion value denotes a better model fit [32].

#### Fixed and random effects.

Adjusted odds ratios (aORs) along with their 95% confidence interval (CI) were used to report the outcomes of fixed effects (measures of association) of the factors associated with ITN use. To account for the hierarchical structure of our data, we incorporated random effects at the community level (level 2) to capture unexplained variation in ITN use between communities after accounting for measured covariates. The Intra-class Correlation (ICC) and Median Odds Ratio (MOR) were used to quantify the likely contextual effects [33]. With the use of ICC, we assessed the similarity between respondents living in the same community. The ICC is a measure of the clustering of odds of ITN use in the same community, showing the percentage of the total variance in ITN use attributable to community-level factors. The MOR estimated the median increase in odds of ITN use if a woman moved to a community with higher ITN use probability. When MOR equals one, there is no community-level variation; higher MOR values indicate stronger community contextual effects The linear threshold was utilised to compute ICC using the Snijders and Bosker formula [34], while MOR measured the heterogeneity of unexplained clusters. This approach allowed us to distinguish between individual-level variation and community-level influence on ITN use patterns.

#### Ethical consideration.

We hereby confirm that all methods and procedures were performed in accordance with the relevant guidelines. The 2021 NMIS protocol was reviewed and approved by the ICF Institutional Review Board. The protocol was also approved in Nigeria by the National Health Research Ethics Committee of Nigeria (NHREC). Written and verbal consent were obtained from participants prior to the interview. A formal request to analyse the NMIS datasets was made by the authors and authorization was granted by MEASURE Evaluation, the custodian of the datasets. All analyses were performed in anonymized forms. The datasets are available in the public domain via https://dhsprogram.com/data/dataset/Nigeria_MIS_2021.cfm?flag=1.

## Results

**Fig 1:**
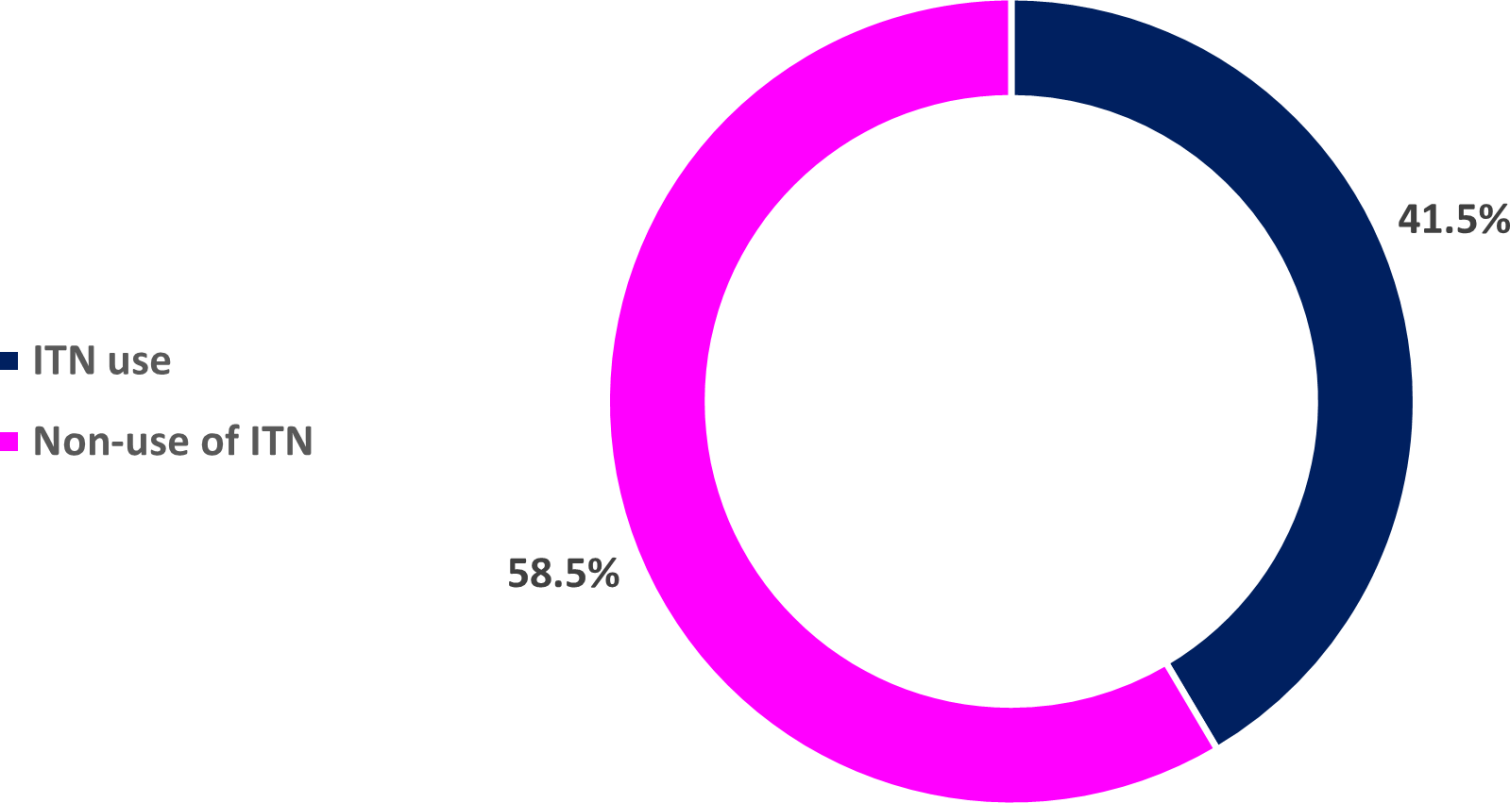
Prevalence of ITN use among women of reproductive age in Nigeria. From Table 1, women with no formal education/primary education (49.7%), Islam (49.4%), have 5+ living children (50.0%), poor (50.5%), from male headed households (43.2%), North East (58.6%), North West (56.7%), rural dwellers (43.8%), from mono-ethnic community (49.1%), from community with low-level of poverty (52.7%) and low-level of education (52.9%) had higher prevalence of ITN use respectively.

The weighted prevalence was 41.5% (95%CI: 39.7%–43.3%). Fig 1 showed that 41.5% of Nigerian women of reproductive age reported ITN use.

**Table 1:** Prevalence of ITN use among Nigerian women of reproductive age (n= 14,476).

Variable	n (%)	Prevalence of ITN use, % (95% CI)	P
Age (in years)			0.205
15–24	5129 (35.4)	41.3 (38.9–43.7)	
25–34	5027 (34.7)	42.6 (40.5–44.8)	
35–49	4320 (29.8)	40.4 (38.2–42.7)	
Education			<0.001
No education/primary	6769 (46.8)	49.7 (47.1–52.3)	
Secondary/higher	7707 (53.2)	33.3 (31.2–35.4)	
Religion			<0.001
Christianity	7058 (48.8)	30.5 (28.1–33.0)	
Islam	7344 (50.7)	49.4 (46.7–52.0)	
Others	74 (0.5)	19.7 (11.4–32.0)	
Exposed to malaria messages			0.739
No	7720 (53.3)	41.3 (38.9–43.6)	
Yes	6756 (46.7)	41.8 (39.5–44.1)	
Number of living children			<0.001
0	4250 (29.4)	33.4 (31.1–35.9)	
1–2	3662 (25.3)	42.1 (39.9–44.4)	
3–4	3479 (24.0)	42.7 (40.3–45.2)	
5+	3085 (21.3)	50.0 (47.1–52.8)	
Wealth			<0.001
Poor	5052 (34.9)	50.5 (47.8–53.3)	
Non-poor	9424 (65.1)	36.1 (34.0–38.3)	
Sex of household head			<0.001
Male	12339 (85.2)	43.2 (41.3–45.2)	
Female	2137 (14.8)	30.0 (27.1–33.2)	
Region			<0.001
North Central	2674 (18.5)	33.6 (29.5–38.0)	
North East	2523 (17.4)	58.6 (54.3–62.7)	
North West	3635 (25.1)	56.7 (53.2–60.1)	
South East	1523 (10.5)	21.1 (17.2–25.6)	
South South	2148 (14.8)	20.3 (17.5–23.4)	
South West	1973 (13.6)	23.6 (20.3–27.4)	
Place of residence			<0.001
Urban	4930 (34.1)	36.6 (33.9–39.4)	
Rural	9546 (65.9)	43.8 (41.5–46.2)	
Community-level ethnic			<0.001
Mono-ethnic	3568 (24.7)	49.1 (44.8–53.4)	
Multi-ethnic	10908 (75.3)	39.1 (36.9–41.4)	
Community-level poverty			<0.001
Low	4934 (34.1)	52.7 (49.3–56.1)	
Medium	4779 (33.0)	45.0 (41.0–49.1)	
High	4763 (32.9)	24.0 (21.0–27.2)	
Community-level education			<0.001
Low	4857 (33.6)	52.9 (49.4–56.4)	
Medium	4839 (33.4)	41.7 (38.0–45.4)	
High	4780 (33.0)	26.3 (22.8–30.2)	
Community-level exposure to malaria messages			0.221
Low	4850 (33.5)	44.1 (40.3–48.0)	
Medium	4815 (33.3)	41.4 (37.7–45.2)	
High	4811 (33.2)	38.8 (34.8–43.0)	

### Measures of variations (random effects) and model fit statistics

In Table 2, Model IV (full model) was selected as the most suitable based on the AIC and BIC values (16054.32 and 16213.50 respectively), where lower values indicate better model fit. The variations in the odds of ITN use communities (σ^2^ = 1.04) was estimated. Results from Median Odds Ratio became the evidence of community contextual factors shaping ITN use. It was estimated that if a women moved to another community with a higher probability of ITN use, the median increase in their odds would be 2.64 with ICC of 24.0%. At community level, the explained variance was 49.3%. This implied that a good amount of variances in ITN use has been explained by the community-level factors. PCV helped in understanding the contribution of added covariates to reducing unexplained variance. A higher PCV indicated that the predictors in the model are explaining a larger proportion of the variance at the community-level.

**Table 2:** Random effect estimates of individual and community-level factors associated with ITN use.

Random-effect	Model I	Model II	Model III	Model IV
**Community-level**				
Variance (95% CI)	2.05 (1.76–2.39)*	1.88 (1.60–2.22)*	1.02 (0.86–1.20)	1.04 (0.88–1.23)*
Explained variance (PCV)	Reference	8.3%	50.2%	49.3%
MOR	3.92	3.70	2.61	2.64
ICC	38.4% (34.9–42.1%)	36.4% (32.7–40.3%)	23.6% (20.7–26.7%)	24.0% (21.1–27.2%)
**Model fit statistics**				
AIC	16481.23	16353.59	16188.34	16054.32
BIC	16496.39	16429.39	16286.88	16213.50
Log-likelihood	-8238.62	-8166.80	-8081.17	-8006.16
**Sample size**				
Individual	14476	14476	14476	14476
Community	567	567	567	567

Model I – baseline model with no explanatory variables, or empty null model (unconditional model).

Model II – solely taking into account individual-level factors.

Model III – solely taking into account community-level factors.

Model IV – full model adjusted for characteristics at the individual, household, and community levels.

AIC Akaike’s Information Criterion, BIC Bayesian Information Criterion, PCV Proportional Change in Variance, ICC Intra-class correlation.

*Significant at p < 0.05.

### Measures of associations (fixed effects)

Results from Table 3 showed Muslim women had 21% (aOR= 0.79; 95% CI: 0.67–0.94) lower odds of ITN use, when compared with Christian women. Those who had living children were more likely to report ITN use when compared with women with no child. The non-poor women had higher odds of ITN use when compared with poor women (aOR= 1.35; 95% CI: 1.19–1.52). In addition, women from multi-ethnic had 26% lower odds of ITN use, when compared with those from mono-ethnic community (aOR= 0.74; 95% CI: 0.57–0.94). The geographical region was significantly associated with ITN use among Nigerian women. Those from high-level community poverty had 53% reduction in ITN use when compared with women from low-level community poverty (aOR= 0.47; 95% CI: 0.32–0.68).

**Table 3:** Fixed effect of individual and community-level factors associated with ITN use.

Variable	Odds ratio (95% CI)			
	Model I	Model II	Model III	Model IV
Education				
No education/primary		1.00		1.00
Secondary/higher		0.93 (0.83–1.04)		1.06 (0.94–1.19)
Religion				
Christianity		1.00		1.00
Islam		1.31 (1.11–1.54)*		0.79 (0.67–0.94)*
Others		0.52 (0.25–1.09)		0.51 (0.24–1.06)
Number of living children				
0		1.00		1.00
1–2		1.59 (1.47–1.77)*		1.64 (1.46–1.83)*
3–4		1.53 (1.37–1.72)*		1.60 (1.42–1.79)*
5+		1.67 (1.48–1.89)*		1.73 (1.53–1.95)*
Wealth				
Poor		1.00		1.00
Non-poor		1.13 (1.00–1.27)		1.35 (1.19–1.52)*
Sex of household head				
Male		1.00		1.00
Female		0.89 (0.78–1.01)		0.95 (0.84–1.08)
Region				
North Central			1.00	1.00
North East			3.67 (2.61–5.16)*	4.03 (2.85–5.71)*
North West			3.00 (2.18–4.14)*	3.33 (2.40–4.63)*
South East			0.33 (0.22–0.49)*	0.29 (0.19–0.44)*
South South			0.61 (0.43–0.85)*	0.55 (0.39–0.77)*
South West			0.66 (0.47–0.93)*	0.63 (0.44–0.89)*
Place of residence				
Urban			1.00	1.00
Rural			1.34 (1.06–1.71)*	1.28 (0.99–1.63)
Community-level ethnic				
Mono-ethnic			1.00	1.00
Multi-ethnic			0.74 (0.58–0.95)*	0.74 (0.57–0.94)*
Community-level poverty				
Low			1.00	1.00
Medium			1.19 (0.89–1.59)	1.03 (0.76–1.39)
High			0.56 (0.39–0.80)*	0.47 (0.32–0.68)*
Community-level education				
Low			1.00	1.00
Medium			1.43 (1.04–1.96)*	1.34 (0.97–1.87)
High			1.27 (0.83–1.92)	1.17 (0.76–1.81)

Model I – baseline model with no explanatory variables, or empty null model (unconditional model).

Model II – solely taking into account individual-level factors.

Model III – solely taking into account community-level factors.

Model IV – full model adjusted for characteristics at the individual, household, and community levels.

*Significant at p < 0.05.

## Discussion

Our study found that the prevalence of ITN use among women of reproductive age in Nigeria was 41.5% (95% CI: 39.7, 43.3). This figure, while showing an improvement from the 36% reported in the 2018 NDHS [15], still falls short of the national target of 80% utilization by 2025 [10]. The prevalence we observed is consistent with other studies in similar settings, such as the 43.2% ITN use reported among adults in northern Ethiopia [35]. However, it is notably higher than the 33% reported for the general population in sub-Saharan Africa [1], suggesting that women of reproductive age may be more likely to use ITNs compared to some other demographic groups. This difference could be attributed to targeted interventions focusing on maternal health and increased risk perception in this population [36]. Nevertheless, the fact that less than half of the women of reproductive age in Nigeria use ITNs highlights the urgent need for intensified efforts to improve utilization rates.

Socioeconomic and demographic factors emerged as significant predictors of ITN use in our study [37]. Wealth status played a crucial role, with non-poor women having 35% higher odds of using ITNs compared to their poor counterparts. This finding aligns with recent studies that found a positive association between household wealth and ITN use across multiple African countries [38–40]. The influence of wealth on ITN use persists despite free distribution campaigns, suggesting that other costs or barriers associated with ITN use may still be prohibitive for poorer households [25,41]. This association could be explained by the increased awareness of malaria prevention strategies and better interpretation of health messages among more rich households. Interestingly, our study found that women with children were more likely to use ITNs, with odds increasing with the number of living children. This pattern has been observed in recent studies from Mali [42] and Tanzania [43] and may reflect a heightened sense of responsibility and increased risk perception among mothers [44].

Religious affiliation and ethnic diversity have emerged as significant belief and cultural factors influencing ITN use. Muslim women had 21% lower odds of ITN use compared to Christian women, a finding that echoes recent research in Nigeria [45]. This disparity might be rooted in differing health beliefs, social norms, or access to health information across religious groups [46]. The lower likelihood of ITN use in multi-ethnic communities compared to mono-ethnic ones (OR = 0.74, 95% CI [0.57, 0.94]) is a novel finding that warrants further investigation. This may reflect challenges in implementing uniform health interventions in diverse communities or indicate varying cultural attitudes towards ITN use [47,48]. Regional differences were stark, with women in the North East and North West having significantly higher odds of ITN use compared to those in the North Central region. These regional disparities, also noted in recent studies, underscore the need for tailored, context-specific interventions that account for local sociocultural factors and malaria transmission intensity [17,19].

Community-level factors played a substantial role in determining ITN use, accounting for 24% of the total variance in the model. This finding aligns with recent multi-country analyses that have highlighted the importance of community context in health behaviors [23]. Notably, women in communities with high poverty levels had 53% lower odds of using ITNs compared to those in low-poverty communities, even after controlling for individual wealth status. This suggests that community economic conditions may influence ITN use through mechanisms beyond individual purchasing power, such as the overall infrastructure, access to health services, or community-wide health education [49]. Interestingly, community education level did not show a significant association with ITN use, in contrast to some recent studies [39]. This discrepancy might be due to the strong influence of other community-level factors in our model or reflect the complex interplay between education and other socio-economic variables at the community level [50]. The substantial community-level variation observed in our study (ICC = 0.24, MOR = 2.64) emphasizes the need for community-focused interventions to complement individual-targeted strategies to promote ITN use [51].

### Strengths and limitations

This study had several strengths and limitations that warrant consideration. A key strength is the use of NMIS dataset, which provides a large, nationally representative sample, allowing for robust statistical analyses and generalizability of findings to women of reproductive age across Nigeria. The multilevel analytical approach employed in this study is another strength as it accounts for the hierarchical structure of the data and allows for the examination of both individual- and community-level factors influencing ITN use. The inclusion of a wide range of socio-demographic, cultural, and community-level variables provides a comprehensive understanding of the factors associated with ITN use. However, this study had some limitations. The cross-sectional nature of NMIS data precludes the establishment of causal relationships between the identified factors and ITN use. There may be recall bias in self-reported ITN use, potentially leading to over- or underestimation of actual usage. Furthermore, while the NMIS provides extensive information, it may not capture all relevant factors influencing ITN use such as detailed information on malaria knowledge, risk perception, or specific cultural beliefs.

## Conclusion

The findings revealed that despite progress, ITN utilization remains suboptimal, with less than half of the target population using ITNs. Our epidemiological analysis has identified key individual and community-level factors associated with lower ITN use, including religious affiliation (aOR = 0.79 for Muslim women), community poverty (aOR = 0.47 for high-poverty communities), and ethnic diversity. The substantial impact of community-level factors (ICC = 24.0%, MOR = 2.64) points to important contextual influences that require further investigation. While our findings establish which populations have lower ITN utilization, we acknowledge that understanding the specific mechanisms behind these associations requires additional research, particularly qualitative investigations to explore why these disparities exist and what specific cultural, social, and structural barriers affect ITN use in different contexts. To achieve the national target of 80% ITN utilization by 2025, future research should focus on examining the specific barriers and facilitators of ITN use within identified high-risk groups, thereby informing more effective, evidence-based interventions that address both the practical and cultural aspects of ITN utilization among women of reproductive age in Nigeria. By addressing these multifaceted determinants, policymakers and health practitioners can develop more effective strategies to increase ITN use, ultimately contributing to the reduction of malaria burden among women of reproductive age in Nigeria, and potentially in similar settings across sub-Saharan Africa.
